# Efectos del etiquetado nutricional frontal de alimentos y bebidas: sinopsis de revisiones sistemáticas

**DOI:** 10.26633/RPSP.2019.62

**Published:** 2019-08-12

**Authors:** Gabriela Santos-Antonio, Fernando Bravo-Rebatta, Patricia Velarde-Delgado, Adolfo Aramburu

**Affiliations:** 1 Centro Nacional de Alimentación y Nutrición Centro Nacional de Alimentación y Nutrición Instituto Nacional de Salud Lima Perú Centro Nacional de Alimentación y Nutrición, Instituto Nacional de Salud, Lima, Perú.

**Keywords:** Etiquetado nutricional, comportamiento del consumidor, estado nutricional, literatura de revisión como asunto, Nutritional labeling, consumer behavior, nutritional status, review literature as topic, Rotulagem nutricional, comportamento do consumidor, estado nutricional, literatura de revisão como assunto

## Abstract

**Objetivos.:**

Sintetizar la información disponible sobre el efecto del etiquetado nutricional frontal en la elección, compra y consumo de alimentos y bebidas, y el estado nutricional de los consumidores, e identificar los factores que influyen en su efectividad.

**Métodos.:**

Se realizó una sinopsis de revisiones sistemáticas (RS) conforme a las recomendaciones PRISMA. La búsqueda bibliográfica se realizó en Medline (Pubmed), The Cochrane Library, LILACS, EBSCOhost y Scopus, limitada a estudios publicados en español o inglés sin restricción por fecha de publicación. La calidad metodológica se evaluó utilizando la herramienta AMSTAR 2.

**Resultados.:**

Se incluyeron siete RS. El etiquetado frontal facilitó la elección de alimentos saludables y tuvo un efecto variable sobre las dimensiones de consumo y compra. Ninguna RS evaluó el efecto sobre el estado nutricional. El costo y sabor, los hábitos alimentarios, el nivel educativo y los sistemas dominantes de procesamiento de información en el consumidor influyeron en su efectividad. La mayoría de RS mostraron limitaciones metodológicas y un nivel de confianza críticamente bajo.

**Conclusiones.:**

El etiquetado frontal tuvo efecto positivo en la elección de alimentos saludables, con resultados variables en las dimensiones de compra y consumo. Se necesitan estudios locales con una adecuada calidad metodológica para identificar el formato de etiquetado más efectivo en cada país. Su implementación como política de salud pública debe acompañarse de estrategias para mejorar el acceso a alimentos saludables, promover la actividad física y brindar educación nutricional a los consumidores.

## Abstract (en)

**Objectives.:**

Synthesize available information on the effects of front-of-package nutritional labeling on choice, purchase, and consumption of food and beverages, and nutritional status of consumers, and identify factors that influence its effectiveness.

**Methods.:**

A synopsis of systematic reviews was carried out following PRISMA recommendations. A literature search was performed in MEDLINE (PubMed), Cochrane Library, LILACS, EBSCOhost, and Scopus, limited to studies published in Spanish or English with no restrictions on date of publication. Methodological quality was evaluated using AMSTAR 2.

**Results.:**

Seven systematic reviews were included. Front-of-package labeling facilitated healthy food choices and had a variable effect on consumption and purchase dimensions. No systematic review evaluated effects on nutritional status. Cost and flavor, eating habits, educational level, and dominant information processing systems in the consumer influenced labeling effectiveness. Most of the systematic reviews showed methodological limitations and critically low confidence levels.

**Conclusions.:**

Front-of-package labeling had a positive effect on healthy food choices, with variable results on purchase and consumption dimensions. Local studies with adequate methodological quality are needed to identify the most effective labeling format in each country. Implementation of labeling as public health policy should be accompanied by strategies to improve access to healthy foods, promote physical activity, and provide nutrition education to consumers.

## RESUMO

**Objetivos.:**

Sumarizar os dados disponíveis referentes ao efeito da rotulagem nutricional frontal na escolha, compra e consumo de alimentos e bebidas e no estado nutricional dos consumidores e identificar os fatores que influenciam a efetividade desta medida.

**Métodos.:**

Foi realizado um sumário de estudos de revisão sistemática segundo as recomendações PRISMA. Foi feita uma busca da literatura nas bases de dados MEDLINE (PubMed), Cochrane Library, LILACS, EBSCOhost e Scopus de estudos publicados em espanhol ou inglês, sem restrição de data de publicação. A qualidade metodológica foi avaliada com o uso da ferramenta AMSTAR 2.

**Resultados.:**

Sete estudos de revisão sistemática foram selecionados. A rotulagem frontal facilitou a escolha de alimentos saudáveis e teve um efeito variável nas dimensões de consumo e compra. Nenhum estudo avaliou o efeito no estado nutricional. Custo e sabor, hábitos alimentares, nível educacional e sistemas dominantes de processamento de informação do consumidor tiveram influência na efetividade desta medida. Os estudos de revisão apresentaram, na sua maioria, limitações metodológicas e um grau de confiança criticamente baixo.

**Conclusões.:**

A rotulagem nutricional frontal teve efeito positivo na escolha de alimentos saudáveis, com resultados variáveis nas dimensões de compra e consumo. É preciso realizar estudos locais com qualidade metodológica adequada para identificar o formato de rotulagem mais efetivo em cada país. A implementação desta medida como política de saúde pública deve ser acompanhada por estratégias para melhorar o acesso a alimentos saudáveis, promover a atividade física e proporcionar educação nutricional aos consumidores.

El sobrepeso y la obesidad afectan a más de un tercio de la población mundial (1) y su frecuencia muestra un rápido aumento en los países de bajos y medianos ingresos (2). En América Latina, se estima que 360 millones de personas (58% de la población) presentan sobrepeso u obesidad (3), un problema de salud que aumenta la mortalidad global (1), el riesgo de enfermedades no transmisibles (1,4–7), afecta a la salud mental (8) y a la calidad de vida (9), y genera importantes cargas económicas para la sociedad y los sistemas de salud (10,11).

La alimentación no saludable representa uno de los principales factores contribuyentes al desarrollo de sobrepeso, obesidad y enfermedades no transmisibles (12). Los patrones alimentarios han cambiado sustancialmente en las últimas décadas (13). Así, se observa un aumento de la disponibilidad de alimentos y calorías *per cápita* en la mayoría de países (14,15) y del consumo de alimentos ultraprocesados ricos en azúcares y grasas saturadas, que constituyen en la actualidad una de las principales fuentes de calorías de la población (16).

El etiquetado nutricional de alimentos y bebidas industrializados tradicionalmente ha proporcionado información sobre ingredientes y nutrientes en la parte posterior o lateral de los productos (17) y está regulado a escala internacional por la Comisión del Códex Alimetarius (18). De forma complementaria, en algunos países de América Latina, como Ecuador, México, Chile y Perú, el etiquetado frontal resumido y simplificado sobre nutrientes relevantes para la salud es obligatorio (19) y se ha propuesto como una estrategia costo-efectiva (20) para mejorar la calidad de la alimentación, empoderar a los consumidores y facilitar la elección y el consumo de alimentos saludables (21).

El formato y la información presentada en el etiquetado frontal varían notablemente y pueden generar confusión en los consumidores (19). Asimismo, existen controversias respecto a la influencia de factores que podrían limitar su efectividad, como el entorno en que las personas compran sus alimentos (22), las expectativas sobre el sabor (23), el precio (24), el contexto cultural (25), el nivel socioeconómico (26), el género, la edad (27) o la predisposición para adoptar una alimentación saludable (17).

El presente estudio tiene como objetivo sintetizar la información disponible sobre el efecto del etiquetado nutricional frontal en la elección, compra y consumo de alimentos y bebidas y el estado nutricional de los consumidores, e identificar los factores que influyen en su efectividad.

## MATERIALES Y MÉTODOS

Este estudio se realizó siguiendo las recomendaciones de la declaración PRISMA para revisiones sistemáticas (28). El protocolo del estudio fue aprobado por la Dirección Ejecutiva de Prevención del Riesgo y Daño Nutricional del Centro Nacional de Alimentación y Nutrición de Perú (está disponible previa solicitud a los autores).

Se consideraron elegibles los estudios que cumplieron los siguientes criterios: 1) población: general, aparentemente sana; 2) intervención: etiquetado nutricional frontal, incluyendo sistemas de nutrientes específicos (por ej., guías diarias de alimentación, semáforo nutricional, octágonos nutricionales), sistemas de resumen (sellos o logos), y sistemas de información por grupo de alimento (declaraciones de propiedades de salud o nutrición); 3) desenlaces: elección, compra, consumo de alimentos, estado nutricional y factores que influyen en la efectividad del etiquetado nutricional frontal, y 4) diseño de los estudios: revisiones sistemáticas de ensayos clínicos o estudios observacionales.

La búsqueda bibliográfica se realizó en MEDLINE (Pubmed), The Cochrane Library, LILACS, EBSCOhost y Scopus. Además, se llevó a cabo una búsqueda manual en Google Scholar y se verificaron las listas de referencias de los estudios identificados, con la finalidad de incluir cualquier referencia adicional relevante. La fecha de la última búsqueda fue el 13 de setiembre de 2018. (La estrategia de búsqueda completa se encuentra disponible previa solicitud a los autores.)

Los registros obtenidos en la búsqueda sistemática se evaluaron revisando los títulos y los resúmenes y, a continuación, el texto completo de los artículos seleccionados previamente. La extracción de datos relevantes de los estudios seleccionados se realizó en un formulario elaborado con el programa Microsoft® Excel 2010. La calidad metodológica de los estudios revisados se evaluó con la herramienta AMSTAR 2 (29). Cada fase la realizaron dos revisores de forma independiente. Cualquier desacuerdo se resolvió con debate o consenso del grupo formado por todos los autores.

## RESULTADOS

**FIGURA 1:**
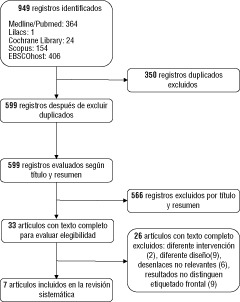
Proceso de selección de los estudios incluidos en la revisión

Con la búsqueda sistemática se identificaron siete revisiones sistemáticas que cumplían los criterios de selección (30–36). La búsqueda en Google Scholar y la revisión de referencias de los estudios incluidos no aportó ningún elemento adicional relevante. El proceso de selección de estudios se muestra en la figura 1.

*Fuente:* Elaboración propia a partir de datos publicados siguiendo las recomendaciones PRISMA.

Todos los estudios se publicaron entre 2013 y 2018. El número de estudios incluidos en cada revisión varió entre 22 y 59. En cuatro revisiones se estudió la población general (30,32,33,36), en una de ellas, adultos (34), y en otra, estudiantes universitarios (31). Cuatro revisiones abordaron específicamente el etiquetado nutricional frontal (32–35), mientras que tres correspondieron a subanálisis del etiquetado nutricional general (30,31,36). El financiamiento en su mayoría procedió de organismos gubernamentales (31,33,35) y de organizaciones sin fines de lucro (32,34) (cuadro 1).

Respecto a la evaluación de la calidad de las revisiones incluidas, una tuvo un nivel de confianza medio (34) y las restantes, críticamente bajo (30–33,35,36). Las debilidades críticas observadas con mayor frecuencia fueron la ausencia de un protocolo previo a la revisión y de una descripción detallada de los estudios excluidos y el riesgo de sesgos en la interpretación o análisis de los resultados.

En el cuadro 2 se presenta un resumen de los principales hallazgos. En cuanto a la elección de alimentos, los semáforos nutricionales facilitaron la elección de alimentos saludables comparados con otros sistemas de etiquetado. En estudios experimentales desarrollados en Australia y Nueva Zelanda se observó que el semáforo nutricional ayudó a los consumidores a seleccionar productos más saludables, mientras que en un ensayo controlado aleatorizado realizado en Alemania se estimó un mayor porcentaje de opciones correctas al tratar de decidir cuál de dos alimentos era más saludable (32).

**CUADRO 1.** Características de los estudios incluidos en la sinopsis

Autor, año	Período de búsqueda	Idiomas incluidos	Estudios incluidos (No.)	Diseño de los estudios	Población	Intervención	Comparación	Desenlaces	Financiamiento
Brown, 2018 (30)	1980 hasta Abril 2016	Inglés	36	Experimentales y cuasi-experimentales	General	EN	No EN	Tamaño de la porción consumida	Ninguno
Christoph, 2018 (31)	Hasta el 18 mayo, 2017	Inglés	22	ECA, cohortes, antes–después	Universitarios	EN	No EN	Dieta y elección de alimentos	Organismo gubernamental
Hawley, 2013 (32)	Enero 2004-Febrero 2011	NN	28	NN	General	ENF	NN	Uso, comprensión, preferencia, percepción o comportamiento	Organización sin fines de lucro
Hersey, 2013 (33)	Enero 1990 – Septiembre 2010	Inglés	38	NN	General	ENF	NN	Atención, procesamiento, comprensión, uso y comportamiento de compra	Organismo gubernamental
Kaur, 2017 (34)	Hasta septiembre 2016	Inglés	31	Experimentales controlados	Adultos	ENF (DNS)	No ENF (DNS)	Decisiones de compra (real o percibida)	Organización sin fines de lucro
Sanjari, 2017 (35)	Enero 1990 - Febrero 2016	Inglés	59	NN	NN	ENF (no DNS)	NN	Factores que influyen en la efectividad	Organismo gubernamental
Sebastian-Ponce, 2015 (36)	Hasta setiembre 2013	Inglés, portugués o español	36	Observacionales	General	EN	Ninguno	Factores asociados a la elección de productos	NN

DNS: declaraciones nutricionales o de salud; ECA: ensayos controlados aleatorizados; EN: etiquetado nutricional; ENF: etiquetado nutricional frontal; NN: no notificado.

*Fuente:* Elaboración propia a partir de datos publicados.

**CUADRO 2.** Resumen de los principales resultados de las revisiones incluidas

Autor, año	Desenlace evaluado	Tipo de etiquetado	Estudios (No.)	Resultados	Conclusión de los autores
Brown, 2018 (30)	Consumo	SN GDA LN	6 6 2	¿? ¿? ¿?	La información nutricional y de salud tuvo un impacto variable en el tamaño de porciones consumidas
Christoph, 2018 (31)	Elección	SN SN, EE, RD	1 3	+ +	ENF tuvo un efecto positivo moderado en la ingesta dietética
Hawley, 2013 (32)	Elección Comprensión Compra Consumo	SN SN SN LN	2 4 2 2	+ ¿? ¿? ¿?	El SN ayudó de manera congruente a identificar productos más saludables
Hersey, 2013 (33)	Atención/procesamiento Comprensión Compra Consumo	EFN EFN EFN EFN	4 7 13 5	+ ¿? ¿? ¿?	EFN con texto simbólico y colores facilitó la interpretación y selección de productos saludables
Kaur, 2017 (34)	Compra	DNS	17	+	DNS tuvo un efecto sustancial en las opciones dietéticas, basado en investigaciones en su mayoría en entornos controlados
Sanjari, 2017 (35)	Efectividad	EFN	59	(*)	El sistema de procesamiento dominante del consumidor influyó en la efectividad del EFN
Sebastian-Ponce, 2015 (36)	Elección	EFN	36	(*)	El precio, lugar de compra/consumo, dimensiones sensoriales, hábitos dietéticos, interpretación de logotipos y educación se asociaron con la elección de productos

+: resultados favorables al etiquetado nutricional; ¿?: resultados mixtos; (*): información presentada de forma narrativa; DNS: declaraciones nutricionales o de salud; EE: equivalentes de ejercicio; EFN: etiquetado frontal nutricional; GDA: guías diarias de alimentación; LN: logos nutricionales; RD: recomendaciones diarias; SN: semáforo nutricional.

***Fuente:*** Elaboración propia a partir de datos publicados.

Un metaanálisis de estudios experimentales sin compra real de alimentos mostró que en los productos que tenían una declaración relacionada con la salud en su etiqueta frontal (“*health claim*”) la probabilidad de ser elegidos fue más alta en comparación con aquellos que no la tenían (OR: 1,75; IC95%: 1,60-1,91). Los mayores efectos se produjeron en las categorías de *“frijoles, legumbres, pescado, huevos, carne y otras proteínas”* (OR: 2,42; IC95%: 1,87-3,12) y *“frutas y verduras”* (OR: 1,92; IC95%: 1,56-2,35) comparados con *“alimentos ricos en grasa y/o azúcar”* (OR: 1,35; IC95%: 1,09-1,60) (34).

Estudios llevados a cabo con estudiantes universitarios muestran que la exposición al etiquetado con semáforos nutricionales redujo las calorías ordenadas y aumentó la probabilidad de compra de alimentos saludables. Asimismo, el etiquetado contextual (semáforos nutricionales, equivalentes de ejercicio o requerimientos diarios sugeridos) fue más efectivo que el etiquetado simple para reducir las calorías ordenadas (-66,9 Kcal; IC95%: -86,7 - -47,2) (31). Del mismo modo, los estudiantes universitarios se mostraron dispuestos a pagar más por un producto con etiquetado nutricional y, en esta categoría, los semáforos nutricionales fueron mejor valorados que las etiquetas nutricionales con información más detallada (32).

Similares hallazgos se observan en la población general. Según otro estudio, los consumidores de los Países Bajos familiarizados con el logo *Choices* (productos con menos sodio, azúcar, grasa saturada, grasa trans, calorías, y mayor nivel de fibra), compraron un mayor número de productos saludables en comparación con aquellos que no estuvieron familiarizados con dicho logo (32).

Por el contrario, los estudios empíricos muestran resultados variables. En uno de ellos, se estimó un aumento significativo de las ventas de productos identificados con tres estrellas del logo *Guiding Stars* (mayor calificación que identifica un alimento como saludable) respecto a aquellos productos que no se identificaron en el programa. En otro estudio, utilizando semáforos nutricionales desarrollado en Reino Unido, no se encontró ningún efecto en las ventas de alimentos saludables, aunque sólo se analizaron dos categorías de alimentos (comidas instantáneas y bocadillos). Por último, en los Estados Unidos de América, en un estudio desarrollado en una tienda minorista seleccionada por conveniencia, no se encontraron diferencias significativas en las ventas de productos saludables (33).

Respecto al consumo de alimentos, los resultados de una revisión sistemática sobre el tamaño de la porción consumida fueron variables (30). En seis estudios incluidos en ella, los semáforos nutricionales produjeron cuatro efectos positivos (disminución del consumo de alimentos densos en energía o pobres en nutrientes en dos estudios, y aumento del consumo de alimentos densos en nutrientes en otros dos estudios), un efecto negativo y tres clasificados como sin efecto. Según otros seis estudios, las Guías Diarias de Alimentación (GDA) produjeron un efecto positivo (disminución del consumo de alimentos densos en energía o pobres en nutrientes), un efecto negativo y cuatro clasificados como sin efecto. En otros dos estudios, no se detectó ningún efecto utilizando los logos *Smart Choices* y *Choices*, en otro el efecto utilizando el logo *Keyhole* fue positivo y en otro, que incluyó el logo de una marca considerada saludable por los autores, no se observó ningún efecto.

Los resultados también varían sobre el consumo notificado y observado. En un estudio no se encontraron diferencias significativas en el consumo real de una torta de chocolate identificada o no con el logo *Choices*, mientras que en otros no se encontraron diferencias respecto al consumo notificado de grasas por consumidores expuestos al etiquetado nutricional frontal (32). Por el contrario, otros estudios concluyen que el etiquetado frontal tuvo un impacto positivo en las dietas y logró disminuir el consumo de grasa y azúcar (33).

No se encontró ninguna revisión sistemática en la cual se evaluara el estado nutricional como desenlace.

Sobre los factores que influyen en la efectividad del etiquetado nutricional frontal, en una revisión sistemática (36) se indicó que los factores determinantes de la elección de alimentos, por encima de la información del etiquetado nutricional, fueron el precio, el sabor del producto, los hábitos dietéticos del consumidor (los conocimientos y creencias fueron utilizados antes que la información nutricional), y la educación (las poblaciones menos favorecidas y con menor educación malinterpretaron con mayor frecuencia la información del etiquetado). El etiquetado frontal, incluyendo semáforos nutricionales y logos, se interpretó con más claridad, aunque algunas declaraciones mostradas en la etiqueta fueron malinterpretadas y en muchas ocasiones consideradas como “licencias” para consumir mayor cantidad de estos productos.

Por otro lado, en otra revisión sistemática los resultados se describen en función del modelo de teoría del proceso dual, que postula que las decisiones pueden tomarse de forma rápida y automática o lenta y deliberadamente (35). Entre sus hallazgos destaca que los consumidores procesan los formatos de etiquetas según su modo de procesamiento dominante, que está influido por variables contextuales y personales. Bajo la presión del tiempo, se refuerza el procesamiento intuitivo y la elección se puede basar en elementos visuales más que en información numérica, mientras que en situaciones de agotamiento cognitivo, como las producidas por la fatiga, el hambre, un número creciente de alternativas o la dificultad para procesar la información presentada limitan la capacidad de codificar señales externas, en cuyo caso los consumidores a menudo recurren a atajos mentales, estrategias de simplificación o reglas empíricas que escapan del análisis racional. El conocimiento sobre nutrición también desempeña un papel importante, y los consumidores con un nivel intermedio de conocimiento son los que hacen elecciones de forma más atenta y con un procesamiento de la información más profundo.

## DISCUSIÓN

En conjunto, los resultados de este estudio indican que el etiquetado nutricional frontal facilitó la elección correcta de alimentos saludables y tuvo un efecto variable sobre las dimensiones de consumo y compra, mientras que factores relacionados con el individuo y su entorno parecen tener una influencia importante en la efectividad de la intervención.

El etiquetado frontal basado en el uso de semáforos nutricionales y declaraciones saludables produjo efectos positivos congruentes sobre la elección de alimentos saludables. Sin embargo, estos resultados proceden principalmente de estudios basados en experimentos de elección, que podrían aislar el efecto de otros factores como el precio o la marca del producto (37). Asimismo, al haberse realizado en condiciones experimentales, su validez externa podría ser limitada (38). Estos factores podrían explicar por qué estos resultados positivos no se apreciaron tan claramente en otras dimensiones, como la compra o el consumo de alimentos saludables.

Las declaraciones saludables ejercieron menor efecto en categorías críticas de alimentos, como alimentos ricos en grasa o azúcar. Esto es congruente con los resultados de estudios que muestran que el uso de etiquetado frontal difiere según la categoría de los alimentos. Por ejemplo, en situaciones como la compra de alimentos poco saludables, se ha señalado que en los consumidores la probabilidad de leer la información nutricional es menor, porque en estas circunstancias se privilegian los criterios hedónicos (35,39).

El efecto del etiquetado frontal sobre la compra de alimentos fue variable. Un hallazgo relevante es que, a diferencia de los estudios realizados con la población general, en los estudios con estudiantes universitarios el efecto del etiquetado frontal en la valoración, compra real y predisposición a la compra de alimentos saludables es congruente. En estos resultados podría estar influyendo el nivel educativo de los consumidores. A este respecto, en una revisión se sugiere que las intervenciones para mejorar la comprensión y el uso de la información del etiquetado nutricional en los consumidores con menores competencias de lectura, escritura y cálculo podrían ser beneficiosas (40).

Por otro lado, el efecto del etiquetado frontal sobre el consumo de alimentos fue positivo en la mayoría de estudios incluidos. Sin embargo, algunos estudios también muestran un aumento del consumo de alimentos ricos en energía o pobres en nutrientes. Este efecto paradójico se ha explicado por la presencia de sesgos cognitivos, como el sesgo de positividad y el denominado “halo de salud”. El sesgo de positividad se produce cuando los consumidores evalúan los productos más favorablemente como resultado de la presencia de información nutricional en el paquete, en comparación con productos similares que no muestran esta información, independientemente del perfil nutricional del producto, mientras que el “halo de salud” se refiere a la generalización de la información de nutrientes específicos a otros atributos del producto, por ejemplo, suponiendo que un producto bajo en colesterol también sea bajo en grasa. Ambos factores tienen el potencial de alentar a los consumidores a aumentar el consumo de estos alimentos si sobrestiman su valor nutricional (30,37,41).

No se identificaron revisiones sistemáticas que evaluaran el impacto del etiquetado en el estado nutricional o desenlaces de salud. La poca evidencia científica disponible sobre esta relación podría explicarse por la inherente dificultad de realizar investigación experimental a largo plazo sobre los efectos en la mortalidad y morbilidad, que no podrían detectarse en menos de una década (37). En ausencia de pruebas experimentales, los estudios observacionales con modelamiento estadístico muestran que el etiquetado frontal produciría una disminución significativa del peso (42), mejoras sustanciales en el consumo de nutrientes y una reducción moderada del consumo de energía (43) y del riesgo cardiovascular (44). Una limitación de este tipo de estudios es que parten de hipótesis favorables al etiquetado nutricional, que podrían diferir de los resultados mixtos observados en la presente revisión.

Algunos factores individuales y contextuales podrían limitar la efectividad del etiquetado nutricional y deberían tomarse en cuenta al diseñar políticas públicas. El precio de los alimentos influyó en la elección más que el etiquetado nutricional (36). Está bien documentado que las dietas saludables son más costosas que las no saludables (26,45,46), por lo cual la alimentación no saludable es más frecuente en personas con un nivel socioeconómico bajo (26). Las políticas de precios de los alimentos, como impuestos, manipulación de precios o subsidios alimentarios, podrían aumentar el consumo de alimentos saludables (24). Sin embargo, algunos estudios sugieren que este efecto podría ser menor en las personas de bajos ingresos que en las de medianos ingresos (26).

Los hábitos alimentarios también se han identificado como un factor relacionado con la efectividad del etiquetado (36). Decidir qué y cuánto comer es para muchos consumidores un comportamiento basado en hábitos. Existe poca evidencia que respalde el papel del etiquetado para mejorar los hábitos alimentarios de las personas. La motivación de las personas para seguir una dieta sana puede disminuir en un ambiente alimentario abundante en alimentos ricos en energía y pobre en nutrientes. En este entorno, la información del etiquetado compite con otros factores y es difícil esperar que los consumidores actúen de acuerdo con objetivos de salud a largo plazo (37).

Una revisión sistemática aportó información para dilucidar la presencia de mecanismos cognitivos que pueden afectar la efectividad del etiquetado nutricional frontal (35). La profundidad del procesamiento de información de un etiquetado frontal puede variar desde una simple mirada y confianza en información parcial hasta un procesamiento profundo, lo cual podría explicar por qué los formatos de las etiquetas no siempre son efectivos. Su efectividad está influida por los sistemas de procesamiento dominantes, que, a su vez, varían según un conjunto de variables personales y contextuales, como la presión de tiempo, motivación, expectativas, conocimiento nutricional, entorno de compra o agotamiento, lo que puede explicar que una persona pueda preferir diferentes formatos de etiquetas en diferentes situaciones y procesarlos de varias maneras.

Las preferencias de los consumidores son también un factor que debe tenerse presente en la implementación de políticas de etiquetado. A este respecto, en una revisión sistemática se indica que los consumidores preferían mayormente un etiquetado frontal simple, con mayor preferencia por el uso de semáforos nutricionales, que, además, incluyera información del total de calorías por porción en alimentos generalmente consumidos en una sola sesión (33). Asimismo, el uso de porcentajes resultó confuso y pocas personas los consideraron útiles, mientras que la aparición de textos con indicaciones de niveles alto, medio o bajo de un nutriente específico mejoró la comprensión del etiquetado. La investigación sobre las preferencias de los consumidores locales es clave para diseñar un etiquetado efectivo, toda vez que los hallazgos de los estudios se basan en consumidores de países de altos ingresos y podrían no ser extrapolables a la realidad local. Como ejemplo, se puede señalar que estudios desarrollados en Chile y Perú han evaluado el modelo de etiquetado frontal denominado octágono nutricional y sugieren una mayor facilidad de lectura e interpretación (47) y una modificación de las preferencias de compra (48), mientras que en el Ecuador los semáforos nutricionales pueden ser utilizados en la parte posterior, aunque estudios cualitativos señalan que podría dificultar la observación de los consumidores (49).

Las principales fortalezas del presente estudio radican en que el diseño empleado permite ofrecer una visión integradora de los efectos del etiquetado frontal en varias dimensiones, como elección, compra y consumo de alimentos. Además, se ha analizado la influencia de factores que podrían limitar su efectividad, incluida la perspectiva de los mecanismos cognoscitivos del consumidor, lo cual representa una aportación novedosa y relevante para mejorar los procesos de implementación del etiquetado como política pública.

Existen algunas limitaciones de las revisiones sistemáticas y de los estudios primarios incluidos en ellas. La mayoría de estudios se realizaron en entornos controlados, con periodos de seguimiento cortos, con alto grado de heterogeneidad, tanto en el diseño de los estudios, como en los métodos y los participantes estudiados, así como en la ausencia de grupos de comparación, los tipos de alimentos evaluados, la terminología y definiciones empleadas, las formas de medición, la notificación de resultados, y el nivel de confianza críticamente bajo de la mayoría de dichas revisiones.

A ellas se añaden, las limitaciones de esta misma sinopsis. Aún tratándose de un tema de gran actualidad, algunos estudios primarios publicados más recientemente pueden no haberse incluido en las revisiones sistemáticas seleccionadas. Asimismo, la elevada heterogeneidad de las intervenciones evaluadas no permitió estimar cuantitativamente la magnitud de los efectos globales mediante un metanálisis. Por último, cabe señalar que la mayoría de estudios proceden de países desarrollados, lo cual podría limitar la representatividad y generalización de los resultados de esta sinopsis.

Por esta razón, aunque los hallazgos son congruentes en mostrar un efecto positivo del etiquetado frontal sobre la elección de alimentos saludables, se necesitan estudios representativos de la población local con adecuada calidad metodológica para identificar de forma apropiada el modelo de etiquetado más efectivo en cada país.

Finalmente, la existencia de numerosos factores individuales y contextuales que se interrelacionan de manera compleja y limitan la efectividad del etiquetado nutricional obliga a que la implementación de un sistema de etiquetado en los países de América Latina se acompañe de un marco de políticas más amplio, que incluya estrategias para mejorar el acceso a los alimentos saludables, promover la actividad física y ofrecer educación nutricional a los consumidores.

## Contribución de los autores.

PV y GS concibieron la idea del estudio original. AA diseñó las estrategias de búsqueda sistemática de estudios. AA, PV, FB, y GS participaron en la selección, extracción y análisis de datos. AA redactó la versión preliminar del manuscrito. Todos los autores revisaron y aprobaron la versión final.

## Financiación.

El presente estudio fue financiado por el Instituto Nacional de Salud del Perú.

## Declaración.

Las opiniones expresadas en este manuscrito son responsabilidad del autor y no reflejan necesariamente los criterios ni la política de la RPSP/ PAJPH y/o de la OPS.
